# From mechanisms to markers: role of pyroptosis in revolutionizing thyroid cancer care

**DOI:** 10.1038/s41420-025-02741-0

**Published:** 2025-10-06

**Authors:** Ningxin Wang, Yuying Chen, Guhan Luo, Dingcun Luo

**Affiliations:** 1 The Fourth Clinical Medical College, Zhejiang Chinese Medical University, Hangzhou First People’s Hospital, Hangzhou, Zhejiang China; 2 Department of Surgical Oncology, Hangzhou First People’s Hospital, Westlake University School of Medicine, Hangzhou, Zhejiang China

**Keywords:** Thyroid cancer, Inflammasome

## Abstract

The global incidence of thyroid cancer, especially papillary thyroid cancer, has increased in recent decades, making it a significant global health issue. Pyroptosis, an important form of programmed cell death, is characterized by pore formation in the cell membrane, membrane rupture, cell swelling, and the subsequent release of cellular contents. Factors released during this process, such as interleukin-1β and interleukin-18, amplify inflammatory effects and trigger immune activation. Increasing evidence indicates that pyroptosis has either tumour-promoting or tumour-suppressing effects at various stages of tumour progression, which has garnered significant attention and warrants further investigation. Thus, harnessing the tumour-inhibitory effects while mitigating the tumour-promoting effects of pyroptosis represents a promising therapeutic strategy for the clinical management of thyroid cancer. Furthermore, pyroptosis-related genes are significantly correlated with the prognosis of thyroid cancer. Therefore, this review provides an overview of the current research regarding the role of pyroptosis in thyroid cancer, focusing on its mechanisms, therapeutic targets, and predictive biomarkers. These findings highlight the importance of pyroptosis in thyroid cancer and offer valuable insights for the development of innovative treatment strategies and accurate prognostic markers.

## Facts

- Thyroid cancer is the one of the most common endocrine tumour, and its incidence is rapidly increase, attracting more attention.
- Pyroptosis, a well-established form of cell death, plays a dual role in the initiation, progression, and prognosis of thyroid cancer, making it an increasingly important focus in scientific research.
- Exploring the driving factors and upstream signalling pathways of pyroptosis in thyroid cancer to provide important insights into how to induce pyroptosis in cancer cells and hinder cancer progression is essential.

## Introduction

Thyroid cancer (TC) is the most prevalent endocrine malignancy, with an increasing incidence worldwide over the past few decades [1–3]. TC has emerged as the third most prevalent form of cancer in China, accounting for approximately 9.8% of all cancer cases in China [4]. The disease includes four main pathological histological subtypes: papillary thyroid carcinoma (PTC), follicular thyroid carcinoma (FTC), anaplastic thyroid carcinoma (ATC), and medullary thyroid carcinoma (MTC) [2]. Among these, PTC is associated with a favourable prognosis, with a 10-year survival rate ranging from 80% to 95%, whereas ATC is characterised by its aggressive nature, resulting in significantly poorer survival outcomes [5, 6]. The aetiology of TC remains unclear, and current studies suggest that it may be related to various factors, such as gene mutations, radiation exposure, and elevated thyroid stimulating hormone levels [7]. Standard treatments for TC currently include surgical resection, radioactive iodine therapy, drug therapy, targeted therapy, and immunotherapy. While many TC patients have a favourable prognosis, approximately 23–30% of patients experience relapse, which significantly impacts the quality of life of patients [7, 8]. Furthermore, the lack of reliable prognostic markers complicates risk stratification and hinders treatment decision-making for clinicians managing TC.

Pyroptosis is a form of proinflammatory programmed cell death that exerts local and systemic effects by releasing inflammatory mediators and recruiting immune cells. The role of pyroptosis in cancer has been widely investigated. Previous studies have indicated that pyroptosis mainly promotes cancer through the release of many inflammatory cytokines, causing cells and tissues to be continuously exposed to a chronic inflammatory environment, which sharply increases the risk of cancer formation [9]. For example, gasdermin E (GSDME), the key protein associated with pyroptosis, has been shown to play an important role in promoting the development of colitis-associated colorectal cancer [10]. Recently, new studies have demonstrated that pyroptosis plays an increasingly pivotal role in tumour suppression. For example, lobaplatin can induce GSDME-mediated pyroptosis in nasopharyngeal carcinoma cells by activating caspase-3 to achieve therapeutic effects [11]. Furthermore, pyroptosis not only induces the death of tumour cells to achieve tumour suppression but also amplifies this effect by activating cytotoxic T cells, increasing macrophage phagocytosis, and transforming “cold” tumours into “hot” tumours [12, 13].

Therefore, given the dual-edged nature of pyroptosis in relation to cancer, further investigation of this relationship is crucial. This study aims to systematically review and explore the specific mechanisms by which pyroptosis influences TC, considering both its cancer-promoting and cancer-suppressing roles. Additionally, this study explores potential therapeutic strategies targeting pyroptosis for the treatment of TC, thereby offering new insights for clinical management and prognosis.

## TC

**Fig. 1:**
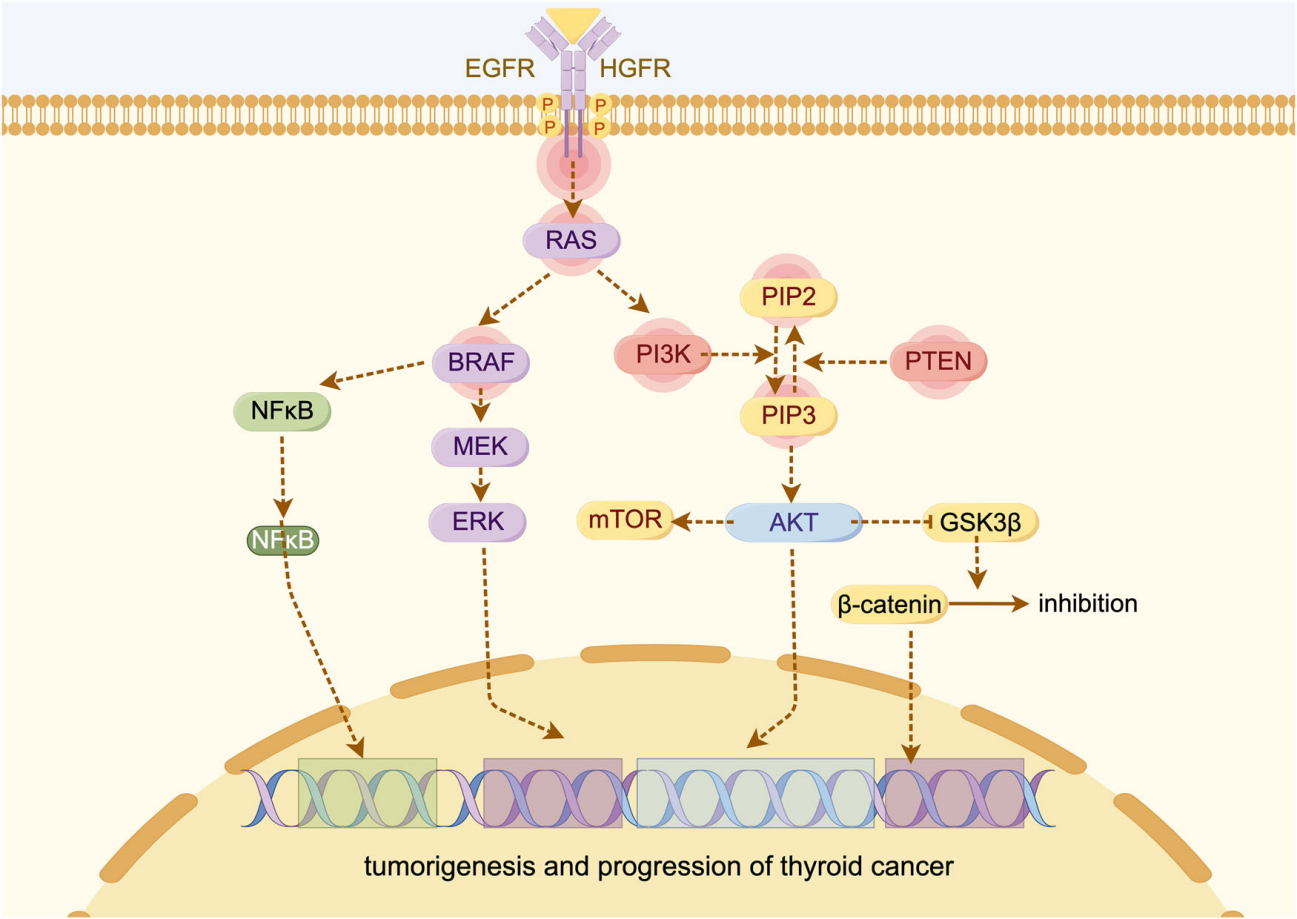
Extracellular signals initiate these pathways by activating receptor tyrosine kinases (RTKs) located on the cell membrane, subsequently leading to the activation of RAS. In the PI3K‒AKT pathway, activated RAS stimulates phosphoinositide 3-kinase (PI3K), which catalyses the conversion of phosphatidylinositol (4,5)-bisphosphate (PIP2) into phosphatidylinositol (3,4,5)-trisphosphate (PIP3). The primary negative regulator of this pathway is phosphatase and tensin homologue (PTEN), which dephosphorylates PIP3 back to PIP2, thereby attenuating downstream signalling. The accumulation of PIP3 promotes the activation of AKT, which translocates to the nucleus and induces the transcription of protumorigenic genes. In the cytoplasm, phosphorylated AKT (p-AKT) further activates downstream effectors, notably the mechanistic target of rapamycin (mTOR) pathway, which facilitates tumorigenesis by increasing mRNA translation. Additionally, p-AKT phosphorylates and inactivates glycogen synthase kinase 3β (GSK3β), a kinase that normally suppresses β-catenin activity. Thus, AKT-mediated inhibition of GSK3β leads to β-catenin stabilisation and nuclear accumulation. In the MAPK pathway, activated RTKs stimulate a signalling cascade involving RAS, RAF (commonly represented by the oncogenic BRAF_V600E mutation), MEK, and extracellular signal-regulated kinase (ERK). Phosphorylated ERK translocates to the nucleus, where it promotes the expression of oncogenes while repressing tumour suppressor genes and those involved in thyroid iodide metabolism.

TC represents one of the most frequently encountered endocrine malignancies in clinical practice. While the majority of patient with TC exhibit indolent biological behaviour and favourable prognoses, significant heterogeneity exists in terms of clinical presentation, progression rates, and long-term outcomes. This variability underscores distinct natural histories across TC subtypes. Advances in molecular genetics and our understanding of tumour evolution mechanisms are progressively transforming the natural history of TC from a ‘black box’ into a predictable and potentially modifiable entity. (Fig. 1). TC originates from thyroid follicular epithelial cells or parafollicular cells (C cells). According to histopathological classification, TC is categorised into PTC, FTC, ATC, and MTC. PTC and FTC originate from thyroid follicular epithelial cells and are collectively known as differentiated thyroid carcinomas (DTCs). The following sections discuss the genetic alterations, natural history, prognosis, and other key aspects of these distinct TC subtypes [14, 15].

### DTCs (including PTCs and FTCs)

PTC, the most prevalent histological subtype of TC, constitutes nearly 85% of all cases of TC and encompasses follicular, diffuse sclerosing, tall cell, and columnar cell subtypes [16]. PTC is characterised by a well-differentiated papillary architecture and diagnostic nuclear features though it is prone to lymphatic metastasis. While the majority of PTC cases are associated with excellent long-term prognoses (survival rates >90%), aggressive variants demonstrating locoregional or distant metastasis, structural recurrence, or progression to high-grade disease may confer significant mortality risk [17].

Genetic alterations involving *RET* rearrangements and point mutations in the *RAS* and *BRAF* proto-oncogenes have been extensively characterised and are present in approximately 70% of PTCs [18], typically in a mutually exclusive pattern. These mutations lead to constitutive activation of the MAPK or PI3K signalling pathways [19]. Specifically, the *RET/PTC* fusion protein retains an intact tyrosine kinase domain, which continuously and aberrantly activates the MAPK cascade, thereby driving the initiation and progression of PTC. Data from The Cancer Genome Atlas (TCGA) revealed that approximately 74.6% of PTCs harbour *BRAF* mutations, with 61.7% specifically harbouring the *BRAF*^*V600E*^ substitution [20]. These findings indicate that the majority of classical PTCs are characterised by the presence of the *BRAF*^*V600E*^ variant [21]. In contrast, the invasive encapsulated follicular variant of PTC is a RAS-like malignancy.

FTC is the second most common histological type of TC and is defined as ‘a malignant epithelial tumour that shows follicular cell differentiation and lacks the diagnostic nuclear features of PTC’. Similar to PTC, distant metastases of FTC predominantly metastasise haematogenously to the lungs and bones, with less frequent involvement of the liver, brain, and other sites [22]. In FTC, *PAX8-PPARG* fusion events are observed in approximately one-third of patients [23]. RAS mutations are commonly detected in FTC (28–68%), the follicular variant of PTC (up to 43%) [24], noninvasive follicular thyroid neoplasms with papillary-like nuclear features (up to 47%) [25], and follicular adenomas (20–25%) [26].

### ATC

The WHO defines ATC as ‘a highly aggressive thyroid malignancy composed of undifferentiated follicular thyroid cells’ characterised by tumour cell infiltration of adjacent thyroid and other tissues, as well as lymphatic and vascular invasion, with a high likelihood of distant metastasis. ATC is a rare but highly lethal form of TC with a historical median survival of approximately 5 months and a 1-year overall survival of 20%. ATC is characterised by aggressive clinical behaviour and a poor prognosis. The most prevalent genetic alterations include *TERT* promoter mutations (detected in 43–73% of patients) and *TP53* mutations (observed in 48–73% of patients) [27]. Notably, *TP53* mutations are highly specific to ATC and are rarely found in differentiated subtypes, underscoring their association with tumour dedifferentiation and an aggressive phenotype.

In *BRAF*-mutated ATCs, cooccurring mutations in *TP53* or *PIK3CA* are frequently observed and are thought to contribute to the dedifferentiation process [28]. In contrast, in ATCs harbouring *RAS* mutations, the molecular mechanisms underlying dedifferentiation remain poorly understood.

### MTC

MTC is a rare malignancy that originates from parafollicular C cells and represents approximately 5% of TCs. Approximately 75% of cases are sporadic, whereas the remaining 25% are hereditary [15]. Activating mutations in the RET proto-oncogene drive tumorigenesis in both contexts. Hereditary MTC is almost universally observed in individuals with multiple endocrine neoplasia type 2 (MEN2) [29], where germline *RET* mutations result in structural changes promoting ligand-independent dimerisation and constitutive activation of the *RET* kinase. In sporadic MTC, mutations in *RET* or *RAS* are frequently identified. Notably, patients with *RAS*-mutated sporadic MTC often exhibit a more indolent disease course and improved prognosis than those harbouring *RET* mutations do [30].

In summary, the molecular pathogenesis of TC is highly heterogeneous across histological subtypes and is driven by distinct oncogenic drivers and signalling pathways that contribute to tumour initiation and progression. Therapeutic targeting of these pathways through their activation or inhibition offers promising avenues for clinical intervention. Emerging evidence suggests that pyroptosis, a form of programmed inflammatory cell death, may play a pivotal role in tumour biology. By investigating the molecular crosstalk between pyroptosis and TC, this review systematically elucidates the potential involvement of pyroptosis in the tumorigenesis, progression, and prognosis of TC. These insights provide a theoretical foundation and future research directions for the development of novel therapeutic strategies aimed at modulating the pyroptosis pathway, offering substantial promise for clinical translation.

## Pyroptosis

**Fig. 2:**
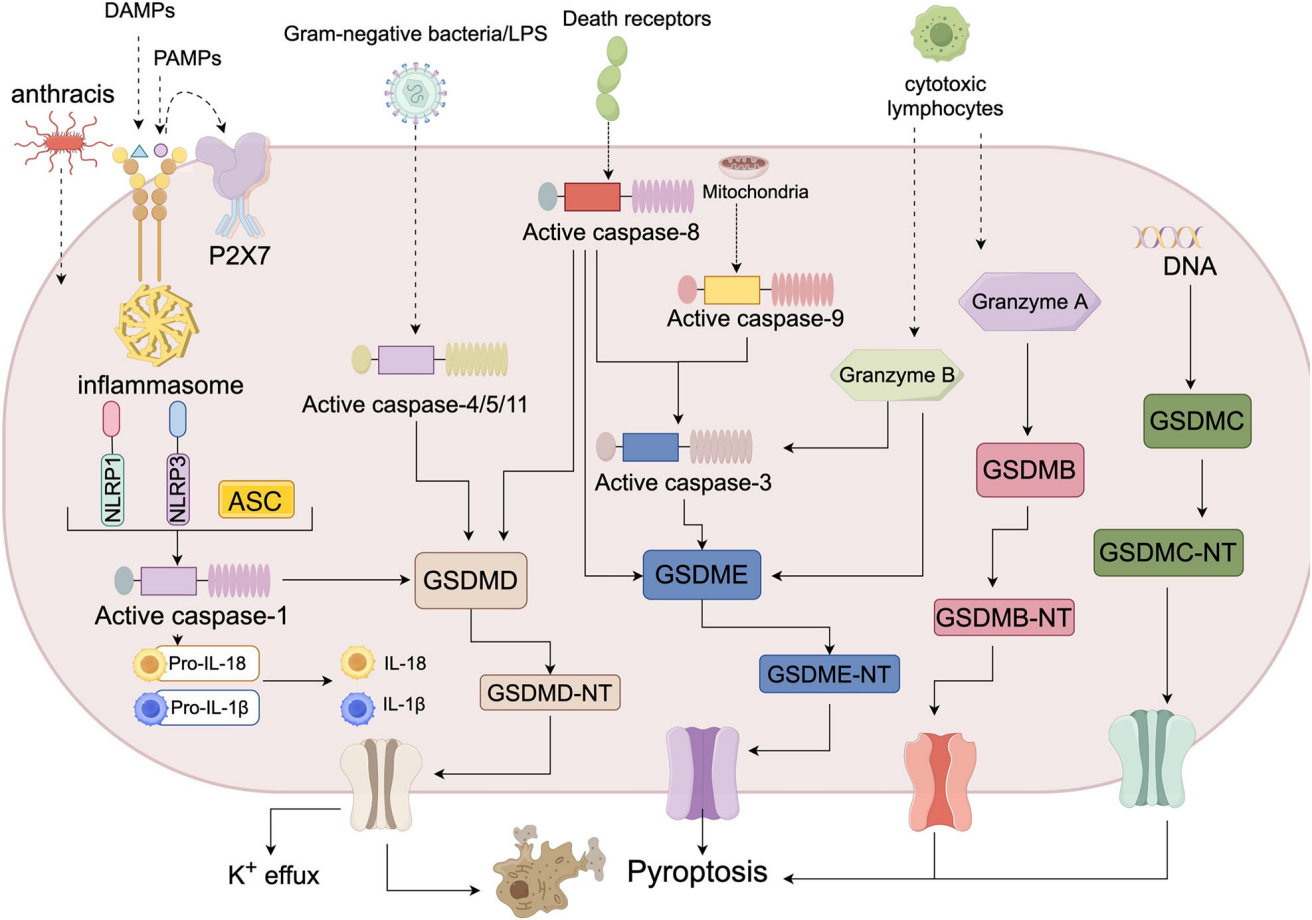
The process and regulatory factors of pyroptosis. Pyroptotic signalling pathways are triggered mainly by the stimulation of damage-associated molecular patterns (DAMPs) and pathogen-associated molecular patterns (PAMPs), leading to the activation of a variety of inflammasome components. The activated inflammasome proteins further activate the cysteine-aspartic specific protease-1 (caspase-1) pathway. Then, activated caspase-1 splits gasdermin D (GSDMD) to produce the N-terminal fragment of GSDMD (GSDMD-NT) and plasma membrane pores, resulting in pyroptosis-dependent cell death. Furthermore, the caspase-1 pathway triggers the formation and release of the inflammatory factors interleukin-1β (IL-1β) and interleukin-18 (IL-18). In addition, lipopolysaccharide (LPS) binds to the caspase-4/5/11 precursor, inducing pyroptosis. caspase-3/GSDME can also cause pyroptosis-mediated cell death. Mitochondrial and death receptors can also trigger the caspase-3 pathway. Activated caspase-3 splits GSDME to produce the N-terminal fragment of GSDME (GSDME-NT), creating plasma membrane pores, cell contraction, and rupture and resulting in pyroptosis-mediated cell death. Granzyme A from cytotoxic lymphocytes and CD8^+^ T cells efficiently cleaves gasdermin B (GSDMB), and granzyme B activates caspase-3 in target cells and then cleaves GSDME. It can also directly cleave GSDME to activate caspase-independent pyroptosis in target cells.

Pyroptosis is an inflammatory form of programmed cell death characterised by pore formation in the plasma membrane, cellular swelling, the release of intracellular contents, chromatin condensation, and DNA fragmentation [31, 32]. In distinct signalling pathways, pyroptosis can be categorised into classical and nonclassical inflammasome pathways, cysteine-aspartic specific protease (caspase)-dependent apoptosis pathways, and granzyme-dependent signalling pathways [33]. In these signalling pathways, upstream caspases or granzymes cleave downstream gasdermin proteins, leading to gasdermin cleavage to induce pyroptosis [34]. The detailed mechanisms of each signalling pathway involved in pyroptosis are described in Fig. 2.

### Classic signalling pathway (caspase-1-dependent pyroptosis)

Classical pyroptosis is triggered primarily by the inflammasome, which subsequently causes the cleavage of GSDMD and the release of various proinflammatory molecules, such as IL-18 and IL-1β. Consequently, the classic signalling pathway, also referred to as the canonical inflammasome pathway, involves intracellular pattern recognition receptors (PRRs), apoptosis-associated speck-like proteins (ASC), and inflammatory caspases [35]. Among these PRRs, the most commonly implicated are nucleotide-binding oligomerization domain-like receptors (NLRs), which include nucleotide-binding oligomerization domain, leucine-rich repeat and pyrin domain-containing 1 (NLRP1), NLRP3, NLRC4, absent in melanoma 2 (AIM2), and pyrin [36–38]. Structurally, NLRP1 consists of an N-terminal pyrin domain (PYD), a nucleotide-binding oligomerization domain (NOD), a leucine-rich repeat, and a C-terminal caspase recruitment domain (CARD) [39]. NLRP1 can be activated by anthrax lethal toxin, muramyl dipeptide and Toxoplasma components [40]. However, unlike that of NLRP1, the C-terminus of NLRP3 lacks a CRAD domain [41–43]. NLRP3 is stimulated by bacteria, viruses, fungi, uric acid, reactive oxygen species (ROS), adenosine triphosphate (ATP), and endogenous damage signals. PRRs have the capacity to recognise pathogen-associated molecular patterns (PAMPs) and damage-associated molecular patterns (DAMPs) [44, 45]. Extracellular ATP triggers IL-1β secretion and caspase-1 activation by activating P2X purinergic receptor 7 (P2X7) and inducing K^+^ efflux [46, 47]. This decrease in the intracellular K^+^ concentration promotes the formation of lysosomes and induces mitochondrial damage [48].

Upon stimulation by PRRs, pro-caspase-1 is recruited either directly through CARD-containing PRRs or indirectly via ASC, which also contains a CARD domain. This recruitment facilitates the assembly of a caspase-1-dependent inflammasome complex [49], allowing caspase-1 to undergo autolytic cleavage and activation. Once activated, caspase-1 can not only cleave the inactive precursors of IL-1β and IL-18 but also process GSDMD, releasing the N-terminal fragment of GSDMD (GSDMD-NT), which forms pores in the cell membrane [50]. IL-18, IL-1β, and other substances are subsequently released into the extracellular space, ultimately triggering inflammation and pyroptosis. Classic inflammasome pathway-mediated pyroptosis mainly occurs in immune cells and serves as a defence mechanism against pathogen infections. Accumulating evidence suggests that inflammasome activation plays a crucial role in cancer progression [51, 52].

### Noncanonical signalling pathway (caspase-4/5/11-dependent pyroptosis)

The noncanonical inflammasome pathway operates independently of the canonical inflammasome complex and is predominantly activated by most gram-negative bacteria [53]. Exogenous lipopolysaccharide (LPS) initiates the expression of type I interferons, which in turn activate their respective receptors, subsequently promoting the expression of caspase-11 [54, 55]. Upon rupture of vacuolar gram-negative bacteria, LPS is released into the cytoplasm. In mice, interferon-inducible guanylate-binding proteins facilitate the detection of this cytosolic LPS, which can directly bind to and activate caspase-11 [56, 57], whereas in humans, intracellular LPS similarly activates caspase-4/5. Although caspase-4/5/11 are unable to cleave IL-1β and IL-18 precursors directly, they cleave GSDMD, which leads to the formation of pores that allow K+ efflux. The loss of intracellular potassium via GSDMD-NT pores triggers the activation of the NLRP3 inflammasome and subsequently caspase-1, thereby driving the maturation and secretion of IL-18 and IL-1β [58, 59]. Moreover, investigations have revealed that LPS stimulation triggers proteolytic cleavage of pannexin-1 channels and subsequent ATP release through a caspase-11-dependent pathway. This molecular cascade leads to the activation of the purinergic receptor P2X7, an ATP-gated ion channel, which facilitates K^+^ efflux in bone marrow-derived macrophages, ultimately culminating in the activation of the NLRP3 inflammasome and caspase-1 [60, 61].

In the noncanonical inflammasome pathway, caspase-4/5/11 activation serves as the molecular initiator that subsequently induces NLRP3 inflammasome assembly and activation. This cascade ultimately leads to proteolytic maturation and secretion of IL-1β and IL-18 while simultaneously mediating pyroptosis.

### Apoptotic caspase-mediated pathway (caspase-3/8-mediated pathway)

In addition to the well-known inflammatory caspases (including caspase-1/4/5/11), emerging evidence has demonstrated that apoptotic initiators and executioner caspases, particularly caspase-8 and caspase-3, respectively, can alternatively induce pyroptosis. The activation of caspase-3 is mechanistically dependent on a positive feedback loop involving the tumour suppressor gene GSDME. Under conditions of elevated GSDME expression, activated caspase-3 cleaves GSDME, releasing its N-terminal fragment. This pore-forming domain subsequently oligomerizes and inserts into the plasma membrane, where it forms transmembrane pores that lead to cell swelling, rupture, and ultimately death [62, 63]. Caspase-8, a critical initiator in apoptotic pathways, also plays a key role in certain pyroptotic processes. Notably, under conditions of caspase-1 deficiency or in the absence of GSDMD, inflammasome complexes can alternatively activate caspase-8, leading to delayed and alternative forms of secondary pyroptosis [44]. Investigative studies revealed that mutation of Cys362 in caspase-8 eliminates its catalytic activity, which is essential for triggering caspase-1-mediated pyroptosis [64, 65]. In addition, the expression of CASP8C362S has the capacity to catalyse ASC spot formation and facilitate caspase-1 activation, thereby promoting the maturation and secretion of IL-1β. These observations demonstrate that the catalytic activity of caspase-8 serves as a molecular scaffold, which is crucial for inflammasome activation and pyroptosis under conditions when both apoptosis and necroptosis are compromised [66, 67]. Furthermore, the death effector domain of caspase-8 directly interacts with the PYD domain of ASC in the presence of caspase-1, thereby enhancing NLRP3 inflammasome assembly and caspase-1 activation, which ultimately drives IL-1β maturation and inflammasome complex formation [68, 69]. In addition, caspase-8 not only directly cleaves GSDMD or GSDME to initiate pyroptosis but also activates caspase-3, which acts as an upstream protein and subsequently cleaves GSDME to regulate pyroptosis [70, 71].

### Granzyme-mediated pathway

Granzymes constitute a family of serine proteases that initiate programmed cell death by cleaving specific intracellular substrates [72]. Recent findings have revealed the pivotal role of granzymes in the induction of pyroptosis. In particular, research has demonstrated that granzyme A, which is released from cytotoxic lymphocytes and CD8^+^ T cells, can effectively cleave GSDMB, leading to the formation of pores in the cell membrane and resulting in cell swelling and rupture, thereby driving pyroptosis [73, 74]. Notably, various splice isoforms of GSDMB exhibit distinct functional properties. Specifically, isoforms 3 and 4 are capable of inducing pyroptosis through N-terminal cleavage, whereas isoforms 1, 2, and 5 do not exhibit this capacity [75]. Research has demonstrated that chimeric antigen receptor T cells can initiate the activation of caspase-3 in target cells through the release of granzyme B, which subsequently cleaves GSDME and results in extensive pyroptosis [76]. Additionally, granzyme B is capable of directly cleaving GSDME at the same site targeted by caspase-3, thereby activating a noncaspase-dependent pyroptosis pathway [77]. Importantly, the direct cleavage of GSDME by granzyme B challenges and expands the existing understanding of pyroptosis induction, suggesting that it may occur through mechanisms beyond those involving caspases.

Through the activation of these four signalling pathways, the pyroptotic process leads to the release of a substantial number of inflammatory cytokines and immune-stimulatory alarmins, including IL-1β, IL-18, ATP, and HMGB1. Among these, IL-1β is secreted through GSDM pores and plays a pivotal role in modulating the tumour microenvironment (TME). IL-1β signalling promotes dendritic cell maturation and drives monocyte differentiation into inflammatory macrophages, thereby facilitating antigen presentation to T cells and enhancing immune-mediated tumour cell elimination. Furthermore, IL-1β directly influences lymphoid cells by stimulating antigen-specific cytotoxic CD8^+^ T-cell responses, expanding Th1 CD4^+^ T-cell populations, and inhibiting the differentiation of immunosuppressive regulatory T cells [78]. This immunological shift transforms ‘cold’ tumours into ‘hot’ tumours, increasing their susceptibility to immune attack. Additionally, GSDME overexpression significantly enhances both the number and functional activity of intratumoral infiltrating NK cells and antigen-specific CD8^+^ T lymphocytes while increasing the phagocytic capacity of tumour-associated macrophages [79]. At this stage, pyroptosis effectively plays a role in tumour immunomodulation.

## Role of pyroptosis-regulated signalling pathways in the pathogenesis and progression of TC

The molecular mechanisms underlying TC are intricate and include a multitude of genetic alterations, aberrant signalling pathways, and dysregulated cellular processes. These factors interact synergistically to drive the initiation, progression, and metastasis of TC [80]. The dual nature of pyroptosis in cancer underscores the need for rigorous investigation into its complex functions within TC. Consequently, this review summarises key pyroptosis-related genes implicated in the pathogenesis of TC, including IL-1β, caspase-1, high mobility group Box 1 (HMGB1), and GSDME, to elucidate their mechanistic interplay and potential contributions to TC biology.

### IL-1β

IL-1β, a key downstream effector of caspase-1, plays a pivotal role in pyroptosis.

Within the classical pathway, caspase-1 activation facilitates the extracellular release of intracellular IL-1β, triggering inflammation and pyroptosis [81]. Given the established link between inflammation and TC initiation and progression, IL-1β, a major inflammatory mediator, significantly influences tumorigenesis. An analysis of various genetic mouse models of breast cancer revealed that the loss of *P53* in cancer cells stimulates IL-1β production by tumour-associated macrophages, thereby promoting systemic inflammation [82]. Furthermore, IL-1β and its genetic polymorphisms are confirmed risk factors for TC [83]. Specifically, elevated serum IL-1β levels, both pre- and postoperatively, are associated with increased IL-1β expression in the tumour tissues of patients with PTC [84]. However, emerging evidence indicates a tumour-suppressive function of IL-1β in TC. IL-1β induces growth arrest and differentiation in MTC cells through MEK/ERK pathway activation via dual-independent signalling mechanisms [85]. Additionally, IL-1β/TNF-α treatment enhances tumour necrosis factor-related apoptosis-inducing ligand (TRAIL)-mediated apoptosis in thyroid cells by increasing TRAIL receptor expression, further supporting its antitumour potential [86].

Therefore, IL-1β, a crucial pyroptosis mediator, plays a dual role in TC progression; however, its specific mechanisms remain to be elucidated. Furthermore, investigating methods to induce and enhance its tumour-suppressive effects represents a promising therapeutic strategy.

### Caspase-1

Caspase-1, also known as IL-1β converting enzyme, was the first mammalian caspase to be discovered [87, 88]. Activated caspase-1 generated upon NLRP3 inflammasome activation cleaves GSDMD to induce pyroptosis and promote inflammatory responses [89].

Mechanistic investigations have demonstrated that AIM2-mediated cell death occurs through a caspase-1-dependent pathway, significantly suppressing tumour proliferation and metastatic progression in *BRAF*-mutant colorectal carcinoma in in vivo models [90]. Membrane-associated RING-CH (MARCH5), a pivotal mitochondrial regulator, has been implicated in the pathogenesis and progression of TC. Mechanistically, MARCH5-mediated downregulation of caspase-1 expression leads to the suppression of pyroptosis, thereby facilitating the progression of TC [91]. Furthermore, TRAIL, a member of the tumour necrosis factor superfamily, has been demonstrated to induce caspase-1-mediated pyroptosis in TC. Specifically, caspase-1 activation under TRAIL stimulation promotes the apoptotic death of TCs [92]. Consequently, caspase-1, a pivotal mediator of pyroptosis, plays a crucial role in suppressing TC progression. The development of therapeutic strategies targeting caspase-1-mediated pathways represents a promising direction for future research in treatment strategies for TC.

### HMGB1

HMGB1 is a highly conserved nuclear protein widely distributed in mammalian cells [93]. HMGB1 mediates pyroptosis through two distinct molecular pathways. First, HMGB1 directly interacts with RAGE on cells, triggering endocytosis and subsequent lysosomal rupture, leading to the release of cathepsin B and the activation of inflammasome formation and caspase-1 [94]. Second, HMGB1 binds to LPS and facilitates its translocation into the cytoplasm of cells, where it activates caspase-11 and downstream caspase-1, thereby inducing pyroptosis [95, 96]. Recent studies have demonstrated that the protein level of HMGB1 is lower in tumour tissues than in adjacent lung tumour tissues, with low HMGB1 expression being significantly associated with lymph node metastasis and extrathyroidal invasion in PTC cells. The *BRAF*^*V600E*^ mutation may downregulate HMGB1 levels through the activation of the mitogen-activated protein kinase signalling pathway [97]. Therefore, increasing the expression of HMGB1 plays an important role in inducing pyroptosis and inhibiting tumour cells in TC, while *BRAF* mutations may block this process.

### GSDME

GSDME, a member of the GSDM family, is cleaved by caspase-3 activation and releases the N-terminal effector domain, which forms a pore in the cell membrane and triggers rapid rupture of the plasma membrane to induce pyroptosis [98]. GSDME-mediated pyroptosis plays a significant role in cancer cell responses to therapeutic agents [99, 100]. For example, in hepatocellular carcinoma (HCC), neobavaisoflavone, a naturally active ingredient isolated from Psoraleae, can induce pyroptosis in HCC cells through the caspase-3-GSDME signalling pathway, providing a basis for the study of new therapies for hepatocellular carcinoma. Similarly, this phenomenon has been reported in TC. In terms of TC, apatinib and melittin synergist [101], Alantolactone (ATL) [102], Prosapogenin A [103] play a therapeutic role by cleaving GSDME and inducing pyroptosis through various ways. These findings strongly suggest that GSDME is a critical mediator in TC and that therapeutic strategies promoting GSDME cleavage represent promising novel approaches for TC treatment.

## Therapeutic regulation of TC via pyroptosis

The standard therapeutic regimen for TC comprises surgical resection, radioactive iodine therapy, and TSH suppression. While most patients with TC exhibit favourable treatment responses, approximately 20% develop recurrence and therapeutic resistance, which adversely affects survival outcomes [7]. Among all types of TC, ATC has the most aggressive clinical behaviour and poorest prognosis. ATC is characterised by rapid progression, with a median survival of 4–6 months and nearly 100% disease-specific mortality, and patients frequently exhibit local tissue invasion, cervical lymph node metastasis, and early distant dissemination [104]. These clinical challenges underscore the critical need for novel targeted therapies. Emerging evidence implicates pyroptosis-related mechanisms as promising therapeutic targets in TC management, with detailed findings summarised in Table 1.

**Table 1:** Summary of drugs that target pyroptosis inhibition in the treatment of thyroid cancer.

Agent	Mechanism	Refs
Ibuprofen	Activation of ASC and NLRP3 and cleavage of GSDMD	[110]
Dabrafenib-Trametinib	Caspase-3 activation and GSDME cleavage were induced, and the release of HMGB1 and IL-1α was induced	[111]
Ruxolitinib	Mitochondrial damage-mediated apoptosis and caspase-3 activation pathway-mediated GSDME-dependent pyroptosis	[124]
Alantolactone	Upregulates ROS mitochondria, activates the caspase-3 pathway and GSDME-dependent pyroptosis	[129]
Prosapogenin A	Active caspase-3/8 and cleave GSDME	[103]
NOD1	Caspase-3-dependent cleavage of PARP and GSDME	[128]
Apatinib and melittin	Caspase-1-GSDMD and caspase-3-GSDME axes simultaneously mediating pyroptosis	[101]

*ASC* apoptosis-associated speck-like protein, *NLRP3* nucleotide-binding oligomerization domain, leucine-rich repeat and pyrin domain-containing 3, *GSDMD* gasdermin D, *Caspase-3* cysteine-aspartic specific protease-3, *GSDME* gasdermin E, *HMGB1* high mobility group Box 1, *NOD1* nucleotide binding oligomerization domain containing 1, *Caspase-1* cysteine-aspartic specific protease-1.

### Ibuprofen

Ibuprofen is a propionic acid derivative that has anti-inflammatory, analgesic, and antipyretic effects [105]. Extensive investigations have demonstrated that ibuprofen can suppress cancer cell proliferation and induce programmed cell death [106–108]. Ibuprofen can be used to treat gastric and prostate cancer by inducing apoptosis and inhibiting the proliferation and metastasis of cancer cells [106, 109]. In ATCs, ibuprofen has been demonstrated to induce pyroptosis through the activation of ASC and NLRP3, as well as the cleavage of GSDMD. This process is further supported by the observation that numerous dying cells exhibit characteristic morphological features of pyroptosis, including bubble-like swelling and membrane rupture [110]. Further studies have shown that thyroid tumour growth in nude mice can be suppressed by ibuprofen-induced pyroptosis in a dose-dependent manner [110]. Therefore, ibuprofen may serve as a promising potential agent for the prevention and treatment of TC, with its mechanism linked to the induction of pyroptosis.

### Dabrafenib-Trametinib (D-T)

The D-T combination represents a clinically established dual-target therapeutic approach consisting of a BRAF inhibitor and a MEK inhibitor and has been extensively utilised in clinical oncology for the treatment of various malignancies [111, 112]. D-T-cell therapy has demonstrated efficacy in treating TC. Specifically, studies have reported that combining D-T therapy with surgery for the treatment of *BRAF*-mutant ATCs can achieve complete remission, prevent recurrence, and lead to a good prognosis [113, 114]. In radioiodine-refractory metastatic differentiated TC with a *BRAF*^*V600E*^ mutation and other variants of *BRAF* mutation with a poorly differentiated phenotype, thyroid tumour cells that underwent D-T therapy showed promising therapeutic effects [115–117]. Further studies demonstrated that D-T primarily exerts its antitumour effect through pyroptosis. Specifically, D-T induces caspase-3 activation and GSDME cleavage and induces the release of HMGB1 and interleukin-1α, collectively promoting pyroptosis [118]. For example, D-T treatment can induce pyroptosis in melanoma cells and significantly improve progression-free survival and overall survival in patients with stage III and IV disease [119]. Collectively, these results indicate that D-T is an effective therapeutic option for cancer suppression by targeting pyroptosis.

### Ruxolitinib (Ruxo)

Ruxolitinib, a JAK inhibitor, has been extensively studied in both preclinical and clinical settings across various tumour types and has demonstrated promising therapeutic efficacy [120]. Current evidence indicates the clinical benefits of Ruxo in treating pancreatic, breast, and ovarian cancers, among other malignancies. Mechanistically, Ruxo primarily suppresses cancer cell proliferation and metastasis by inhibiting the JAK2-STAT signalling pathway [102, 121, 122]. Notably, IKAROS family zinc finger 1 overexpression suppresses the JAK2/STAT5 signalling pathway and promotes pyroptosis, thereby inhibiting the proliferation, migration, and invasion of cancers [123]. Importantly, recent studies have highlighted the significant role of Ruxo in TC. In ATC cells, Ruxo has been shown to induce caspase-3-mediated, GSDME-dependent pyroptosis and downregulate dynamin-related protein 1 (DRP1) expression, thereby contributing to improved prognosis in TC [124]. Collectively, these findings position Ruxo as a promising novel therapeutic agent for TC, largely due to its ability to induce pyroptosis.

### ATL

ATL is a compound derived from the traditional Chinese herbal medicine Inula helenium L. that has significant anti-inflammatory, antibacterial, and antitumour activities [125]. Previous studies have shown that ALT can antagonise various types of cancer cells by inducing apoptosis, inhibiting cell growth and proliferation, and inhibiting metastasis [126, 127]. For example, ALT inhibits the proliferation and induces the apoptosis of breast cancer cells by modulating the protein expression levels of caspase-3 and caspase-12 while also suppressing colony formation and cell migration [128]. However, ALT no longer induces cancer cell death through apoptosis but instead triggers pyroptosis in TC cells. Recent studies have suggested that ATL can induce ATC complicated with apoptosis and GSDME-dependent pyroptosis by increasing the number of ROS in mitochondria and then activating the caspase-3 pathway [129]. However, the underlying mechanism by which ALT induces apoptosis in other cancers but triggers pyroptosis specifically in TC remains unclear. Further elucidation of the therapeutic potential of ALT in TC is warranted.

The above findings underscore the complexity of pyroptosis regulation in TC and highlight several critical knowledge gaps. However, several limitations remain: Overexpression of GSDMD has been shown to significantly inhibit ibuprofen-induced pyroptosis. However, the regulatory mechanisms by which ibuprofen influences GSDMD expression remain unclear. D-T therapy is only effective against *BRAF*-mutated TCs but is limited to *BRAF*-negative TCs. Ruxo has been reported to induce caspase-3-mediated, GSDME-dependent pyroptosis and concurrently downregulate DRP1 expression. Notably, inhibition of the JAK2/STAT3 pathway has also been associated with reduced DRP1 expression [130], suggesting a potential link between these signalling pathways and mitochondrial dynamics. Nevertheless, the precise mechanism by which ALT triggers pyroptosis in TC cells remains to be elucidated. Consequently, addressing these limitations through focused research is imperative for developing effective, personalised therapeutic strategies for TC. Critically, advancing pyroptosis-mediated treatments represents a pivotal future direction.

## Regulation of the prognosis of TC through the pyroptosis-dependent signalling pathway

Certain types of TC, such as ATC, are associated with a poor prognosis. Therefore, early diagnosis and timely intervention are crucial for improving patient outcomes [131, 132]. In recent years, studies have demonstrated that pyroptosis-related genes play crucial roles in predicting the prognosis of TC. Table 2 shows details regarding this.

**Table 2:** Relationships between ferroptosis-related genes and pyroptosis in thyroid cancer.

Ferroptosis related genes	Relationship with pyroptosis	Prognosis of thyroid cancer
CASP6	Promotion	Poor
GSDMC	Promotion	Poor
IL-8	Promotion	Good or poor
H2BC8	Promotion	Poor
CXCL8	Promotion	Poor
PYCARD	Promotion	Poor
SEZ6L2	Promotion	Poor
GJA1	Promotion	Good
TRAF6	Promotion	Good
PRDM1	Promotion	Good
IFI27	Promotion	Good
Siglec-15	Promotion	Poor
PRKACA	Promotion	Poor

*CASP6* cysteine-aspartic specific protease-6, *GSDMC* gasdermin C, *IL-8* interleukin-18, *H2BC8* H2B clustered histone 8, *CXCL8* C-X-C motif chemokine ligand 8, *PYCARD* PYD and CARD domain containing, *SEZ6L2* seizure related 6 homologue like 2, *GJA1* gap junction protein alpha 1, *TRAF6* TNF receptor associated factor 6, *PRDM1* PR/SET domain 1, *IFI27* interferon alpha inducible protein 27, *Siglec-15* sialic acid-binding immunoglobulin-like lectin-15.

### Caspase-6 (CASP6)

Caspase-6 (CASP6), a member of the caspase family, enhances the activation of the caspase11-NLRP3 inflammasome during gram-negative bacterial infections to produce inflammatory cytokines that promote pyroptosis [133]. CASP6 is involved in pyroptosis in breast cancer [134], liver cancer [135], pancreatic cancer [136], and other types of cells. High expression of CASP6 often indicates a poor prognosis of TC, and it is significantly correlated with lymph node metastasis and tumour stage [137]. In related prognostic models, high expression of CASP6 was generally associated with significantly reduced progression-free survival in patients with TC [138]. CASP6 can cleave the transcription factor AP-2α, inactivate it, decrease E-cadherin, increase the expression of matrix metalloproteinase 9, and promote tumour cell migration and metastasis [139]. Therefore, as a pyroptosis-related protein, CASP6 is associated with poor prognosis of TC. Targeting CASP6 inhibition may represent a novel therapeutic strategy to reduce the recurrence of TC.

### GSDMB

GSDMB, a member of the GSDM family, induces pyroptosis upon the activation of granzyme A, the granzyme-mediated pathway mentioned presented in Section “Granzyme-mediated pathway” [140].

Previous studies have shown that GSDM family proteins are abnormally expressed in human cancers and that the overexpression of GSDMB is associated with cancer metastasis [141, 142]. In particular, GSDMB elevation in breast cancer, cervical cancer, and gastric cancer is well documented, and high GSDMB mRNA expression is associated with poor prognosis in patients with breast cancer [143]. Recently, a thyroid prognostic model constructed with pyroptotic genes revealed that high GSDMB levels predict poor TC prognosis, and high expression of GSDMB often indicates tumour metastasis [144, 145]. Therefore, GSDMB, an important pyroptosis protein, can be used as an effective indicator of the prognosis of TC.

### IL-18

IL-18 is a cytokine released at the downstream end of pyroptosis. The key function of IL-18 is to induce the production of INF-γ and mediate Th1 and Th2 immune responses [146]. Many studies have shown that chronic inflammation caused by IL-18 plays an important role in the occurrence and development of tumours [147]. However, in the prognostic risk scoring model for PTC, higher expression of IL-18 is correlated with longer disease-free survival, suggesting that IL-18 may be involved in the suppression of cancer cell growth and that increasing its expression may help improve prognosis [138]. However, the role of IL-18 in TC is controversial and requires further investigation.

### PYD and CARD domain-containing (PYCARD)

PYD and CARD domain-containing (PYCARD), also referred to as ASC, functions as an adaptor protein within the NLRP3 inflammasome and plays a key role in pyroptosis [148]. PYCARDs are widely recognised for their tumour-suppressive role in various malignancies. For example, in gastric cancer, PYCARD expression is significantly higher in normal tissues than in tumour tissues [149], a finding that is also observed in lung cancer [150]. However, in renal clear cell carcinoma, PYCARDs have been shown to promote cell proliferation and migration [151]. In TC, the upregulation of PYCARD expression has been correlated with a poor prognosis [152]. While the specific mechanism of PYCARD in TC requires further investigation, it can be inferred that PYCARD is associated with pyroptosis. Therefore, as a crucial regulator of pyroptosis, PYCARD serves as an important prognostic biomarker in TC.

### Sialic acid-binding immunoglobulin-like lectin-15 (Siglec-15)

Sialic acid-binding immunoglobulin-like lectin-15 (Siglec-15), a novel immunosuppressive molecule, is expressed primarily on various tumour cells and tumour-associated macrophages. Siglec-15 has been shown to inhibit the activation of naive CD8^+^ T cells and facilitate secondary metastasis in breast cancer [153]. Additionally, downregulation of Siglec-15 suppresses the proliferation of osteosarcoma cells [154]. Following Siglec-15 knockdown, the expression of cleaved caspase-3 is elevated. Caspase-3 activation can cleave GSDME, thereby triggering pyroptosis [155]. In the context of TC, high expression of Siglec-15 is associated with immune dysfunction, particularly through the expression of Siglec-15 on immune cells, such as monocytes and macrophages. This expression creates an immune escape environment and is correlated with poor prognosis [156]. Furthermore, targeting Siglec-15 has been proposed as a promising immunotherapeutic approach for anaplastic TC [157]. Thus, elevated levels of Siglec-15 contribute to the inhibition of pyroptosis and facilitate the progression of TC, with high Siglec-15 expression serving as a poor prognostic indicator.

### Protein kinase CAMP-activated catalytic subunit alpha (PRKACA)

Protein kinase CAMP-activated catalytic subunit alpha (PRAKACA), the catalytic subunit α of protein kinase A activated by cAMP, is closely associated with tumour progression. Multiple studies have shown that PRKACA is a pyroptosis-related factor involved in tumorigenesis, development, and the construction of prognostic models [158, 159]. For example, elevated PRKACA transcription can be detected in patients with breast cancer with trastuzumab resistance [160]. In the field of TC, particularly PTC, the mRNA expression of PRKACA is significantly increased in tumour tissues, suggesting that elevated PRKACA levels may serve as indicators of TC, especially PTC [128]. Therefore, PRKACA may be used as a potential cancer biomarker that could facilitate the early diagnosis of TC.

## Pyroptosis and chemotherapy

Studies have demonstrated that chemotherapeutic drugs predominantly induce pyroptosis through GSDME cleavage. In contrast to apoptosis, the presence of GSDME leads to chemotherapy-induced caspase-3 activation, which typically results in pyroptosis as it progresses faster than apoptosis. Although most conventional chemotherapeutic drugs act as apoptosis inducers, cancer cells frequently develop drug resistance by evading apoptotic pathways. Therefore, inducing pyroptosis through nonapoptotic cell death mechanisms represents a promising strategy for overcoming resistance to chemotherapeutic agents.

### Carboplatin

Carboplatin, a well-established chemotherapeutic agent, has been extensively utilised in clinical antitumour therapy and does not exhibit cross-resistance with other antineoplastic agents [161]. Extensive research has demonstrated that carboplatin has good effects on prostate cancer [162], breast cancer [163], and TC. Low-dose carboplatin has been shown to shift the mode of cell death from apoptosis to pyroptosis. In the context of TC, carboplatin can synergistically improve the efficacy of ATC by inducing pyroptosis through caspase-3-, 8-, and 12-dependent pathways. The upregulation of GSDME expression increases the sensitivity of cancer cells to chemotherapeutic drugs and reduces the required dose of chemotherapeutic drugs [164, 165]. Consequently, carboplatin may represent a promising therapeutic strategy for the treatment of TC.

### Programmed death receptor-1/programmed death ligand 1(PD-1/PD-L1)

Programmed death receptor-1 (PD-1) is significantly overexpressed in both tumour cells and tumour-associated antigen-presenting cells [166]. Programmed death ligand 1 (PD-L1), the ligand of PD-1, facilitates the upregulation of GSDMC transcription under hypoxic conditions [167]. Specifically, upon TNFα treatment, GSDMC is cleaved by caspase-8, generating its N-terminal domain, which forms pores in the cell membrane and induces pyroptosis [168]. In HCC, PD-L1 has been shown to upregulate GSDMC expression, which in turn potentiates the invasive, migratory, and proliferative capacities of HCC cells [169]. Increased PD-L1 expression in well-differentiated TCs was first reported in 2013 and was correlated with aggressive clinicopathological features [170, 171]. In PTC, PD-L1 expression is observed in approximately 50% of tumours and is associated with reduced disease-free survival [172]. Furthermore, PD-L1 expression is highly prevalent in poorly differentiated TC specimens [173]. Consequently, immunotherapy targeting the PD-1/PD-L1 pathway may represent a promising therapeutic prospect for advanced, recurrent and metastatic TC.

## Conclusion and outlook

As a distinct form of programmed cell death, pyroptosis critically influences cancer initiation, progression, therapeutic response, and prognosis, making it a major focus of oncology research. Caspase-induced pyroptosis involves cytoplasmic pore formation, cell lysis, membrane denaturation and the secretion of several intracellular components. TC, the most common endocrine malignancy, is rapidly increasing in incidence worldwide and warrants significant clinical attention. To our knowledge, this is the first systematic review synthesising the role of pyroptosis in TC pathogenesis and treatment. This analysis reveals its potential as a novel therapeutic target for advancing TC management.

First, pyroptosis plays a dual role in TC pathogenesis. Paradoxically, while pyroptosis may promote tumorigenesis, immune evasion, angiogenesis, and metastasis, its acute activation results in the recruitment of immunocytes that can suppress tumour progression [174]. Consequently, the therapeutic induction of pyroptosis in TC cells has emerged as a promising anticancer strategy to inhibit proliferation and invasion. Key molecular mediators, including GSDMC and caspase-1, have been confirmed to be closely associated with pyroptosis. However, the context-dependent duality of pyroptosis in TC remains incompletely characterised and warrants systematic investigation. Additionally, the relationships between TC and other core pyroptosis regulators remain underexplored and require more research to understand the pathogenesis of TC comprehensively. Exploring the driving factors and upstream signalling pathways of pyroptosis in TC to provide important insights into how to induce pyroptosis in cancer cells and hinder cancer progression is essential.

Second, a summary of the literature suggests that certain drugs may suppress TC through targeted therapy and immunotherapy, indicating that these drugs could be potential therapeutic targets for TC. Several specific candidate drugs have demonstrated the potential to trigger pyroptosis in cancer cells. However, further research is essential to delineate the underlying mechanisms and develop targeted therapies that modulate inflammasome activation and heat shock protein dysregulation in TC. Moreover, balancing the precise targeting of drugs between normal cells and cancer cells remains a critical research challenge. The tumour-suppressive effects of GSDME are achieved through pyroptosis of cancer cells and the release of inflammatory cytokines, which shift the tumour TME from a ‘cold’ to a ‘hot’ state, significantly enhancing the antitumour activity of CD8^+^ T cytotoxic lymphocytes, macrophages, and tumour-infiltrating NK cells. However, GSDME is highly expressed in normal tissue cells and tumour-infiltrating macrophages, which may exacerbate the toxicity and side effects of chemotherapy [98].

Third, this study revealed a strong correlation between pyroptosis-related genes and the prognosis of TC, highlighting their potential as prognostic biomarkers. These findings provide valuable support for the clinical prognostic evaluation of TC and aid clinicians in formulating treatment strategies. However, the current literature shows that some pyroptosis-related genes associated with TC prognosis are linked to favourable outcomes, whereas others are correlated with poor prognosis, and many predictive genes are primarily identified on the basis of database analyses without experimental validation. Although these genes are related to pyroptosis, further research is needed to determine whether they exert anticancer or procancer effects through pyroptosis, and robust experimental data to clarify the specific mechanisms involved are lacking.
